# Competition for Chiasma Formation Between Identical and Homologous (But Not Identical) Chromosomes in Synthetic Autotetraploids of *Arabidopsis thaliana*

**DOI:** 10.3389/fpls.2018.01924

**Published:** 2019-01-09

**Authors:** Pablo Parra-Nunez, Mónica Pradillo, Juan Luis Santos

**Affiliations:** Department of Genetics, Physiology and Microbiology, Faculty of Biology, Complutense University of Madrid, Madrid, Spain

**Keywords:** *Arabidopsis thaliana*, autotetraploids, chiasma, homologous chromosomes, meiosis

## Abstract

Polyploid organisms provide additional opportunities to study meiosis in a more complex context since more than two potential homologous chromosomes are available. When the chromosome complement of a diploid individual is duplicated, each chromosome is accompanied by one identical and two homologous chromosomes within the same nucleus. In this situation, a competition in pairing/synapsis/chiasma formation between identical and homologous (but not necessarily identical) chromosomes can occur. Several studies have been conducted in different species to address whether there are preferences in crossover formation between identical rather than homologous chromosomes. In this study, multivalent and chiasma frequencies were cytologically analyzed in synthetic autotetraploids of *Arabidopsis thaliana* including the accessions Col, L*er*, and the Col/L*er* hybrid. Fluorescence *in situ* hybridization was conducted to identify each chromosome at metaphase I. The new Col and L*er* tetraploids showed high multivalent frequencies, exceeding the theoretical 66.66% expected on a simple random end-pairing model, thus indicating that there are more than two autonomous synaptic sites per chromosome despite their small size. However, a significant excess of bivalent pairs was found in the Col/L*er* hybrid, mainly due to the contribution of chromosomes 2 and 3. The mean chiasma frequencies of the three artificial autotetraploids were about twofold the corresponding mean cell chiasma frequencies of their diploid counterparts. The relative contribution of each chromosome to the total chiasma frequency was similar in the three genotypes, with the exception of a lower contribution of chromosome 3 in the hybrid. Preferences for chiasma formation between identical and homologous chromosomes were analyzed in Col/L*er* 4x, taking advantage of the cytological differences between the accessions: variations in the size of the 45S rDNA region on the short arm of chromosome 2 and changes in the size and localization of the 5S rDNA region in chromosome 3. We observed a different behavior of chromosomes 2 and 3, i.e., random chiasma formation between identical and homologous chromosomes 2, and preferences for chiasma formation between homologous chromosomes 3. Hence, our results reveal the existence of chromosome-specific mechanisms responsible for these preferences.

## Introduction

Meiosis is a specialized eukaryotic cell division which reduces the number of chromosomes in a parent diploid cell by half to produce haploid gametes. During meiosis, the correct segregation of homologous chromosomes at anaphase I is ensured by the combined action of sister chromatid cohesion and chiasma formation. In many species, chiasmata (the physical attachments between homologous chromosomes) are formed after the recognition of homologous chromosomes (pairing), the close association of paired chromosomes by the synaptonemal complex (SC), and the reciprocal exchange of sequences by the homologous recombination (HR) process.

Polyploids provide additional opportunities to study meiosis in a more complex context since more than two potential partners for these exchanges are available. Depending on their origin, they can show different meiotic behaviors (Sybenga, 1996). Polyploids resulting from the merging of two chromosomal sets from different species (allopolyploids) are expected to show disomic inheritance, with pairs of related chromosomes from the same parental forming preferentially bivalents (Le Comber et al., 2010; Lloyd and Bomblies, 2016). On the other hand, polyploids resulting from within-species duplication events (autopolyploids) generally show tetrasomic inheritance (random synapsis, recombination and segregation of all homologous chromosomes) as a consequence of an extensive multivalent formation (Soltis and Rieseberg, 1986; Wolf et al., 1989; Muthoni et al., 2015; Lloyd and Bomblies, 2016). In this landscape, synapsis and recombination preferences among the members of a tetrasome (set of four homologous chromosomes) can be responsible for cases that present an intermediate behavior between a disomic and a tetrasomic inheritance, and even for the diploidization process (Jannoo et al., 2004; Stift et al., 2008; Meirmans and Van Tienderen, 2013).

The degree of relationship of two chromosomes may be greater than mere homology. For example, when the chromosome complement of a diploid individual is duplicated, each tetrasome is formed by two pairs of completely identical chromosomes; i.e., each chromosome is accompanied by one identical and two homologous chromosomes within the same nucleus. In this landscape, a competition in pairing, synapsis, and recombination between identical and homologous (but not necessarily identical) chromosomes can occur. Attempts to address this issue have been performed mainly in plants since it is very easy to obtain autotetraploids by a colchicine treatment, with only a few examples in animals. Possible preferences between chromosomes of a tetrasome were inferred from analyses to determine the segregation of genetic and/or chromosomal markers (Giraldez and Santos, 1981; Santos et al., 1983; Benavente and Orellana, 1991; Curole and Hedgecock, 2005). The most exhaustive cytological studies were conducted on rye, taking advantage of the existence of C-bands polymorphisms, especially in the nucleolar organizing region (NOR)-bearing chromosome 1R (Orellana and Santos, 1985; Benavente and Orellana, 1989, 1991; Benavente and Sybenga, 2004). In general, in this species there is a trend to identical over homologous preferential associations at metaphase I. This tendency is greater in hybrids with higher chromosomal divergence between the parental diploid plants. This fact indicates chromosome differentiation may play a relevant role in the establishment of such preferences (Benavente and Orellana, 1991; Jenkins and Chatterjee, 1994). These preferences could contribute to the diploidization process of autopolyploids.

In this study, we have analyzed chromosome configurations at metaphase I in autotetraploid meiocytes from the plant model species *Arabidopsis thaliana*. Tetraploid plants were obtained by applying a colchicine treatment to hybrid diploid plants from the cross between Col-0 and L*er*-1 accessions (Col and L*er* onward). We have used the 45S and 5S rDNA sequences as cytological markers. These sequences show quantitative and qualitative variations in chromosomes 2 and 3 of these accessions (Sanchez-Moran et al., 2002). In *Arabidopsis*, the initiation and progression of meiotic recombination is required to establish the SC-mediated pairwise association between homologous chromosomes (Grelon et al., 2001). Therefore, we consider more appropriate the use of the term “chiasma formation preferences” instead of “pairing preferences” throughout this paper. This clarification is necessary because in the mid-20th century and first decade of the current century, in most of the traditional literature on plant cytogenetics, the term chromosome pairing was used as the equivalent of chromosome associations mediated by chiasmata at metaphase I.

## Materials and Methods

### Plant Materials and Growth Condition

Diploid plants of Columbia (Col-0) and Landsberg *erecta* (L*er*-1) accessions (2*n* = 2*x* = 10), and also Col-0/L*er*-1 hybrid plants were treated with colchicine in order to obtain the corresponding autotetraploids (2*n* = 4*x* = 20) (Santos et al., 2003). This treatment consists in applying a 10 μL drop of colchicine at a 0.25% w/v concentration on the center of the plant rosette prior to the first flowering. Seeds from these plants were sown on a mixture of 3 parts of soil and 1 part of vermiculite and grown under constant conditions of 16h day-length, 70% relative humidity and 19°C.

### Cytological Analyses

**FIGURE 1:**
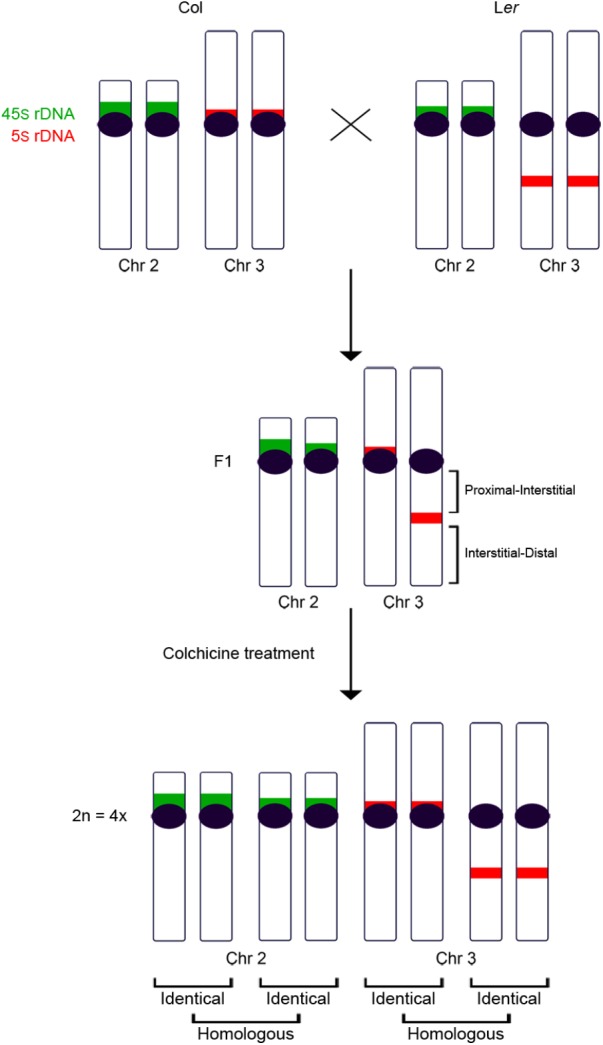
Ideogram of chromosomes 2 and 3 in Col, L*er* and in the hybrid progeny obtained from the cross between both accessions before and after the chromosome duplication. Col and L*er* chromosomes 2 and 3 can be distinguished after applying FISH with 5S (red) and 45S (green) rDNA probes. These differences allow the identification of the parental origin of each chromosome in the hybrid, before and after the colchicine treatment. In the tetraploid hybrid, each chromosome is accompanied by one identical chromosome and two homologous chromosomes.

Fixation of flower buds, slide preparations of pollen mother cells (PMCs), and fluorescence *in situ* hybridization (FISH) were conducted according to Sanchez-Moran et al. (2001), with minor modifications due to the polyploid samples. The DNA probes used comprise ribosomal DNA 45S and 5S loci (Gerlach and Bedbrook, 1979; Campell et al., 1992). The existence of changes in the localization of the 5S rDNA locus belonging to chromosome 3 (Fransz et al., 1998; Sanchez-Moran et al., 2002), and variations in the size of the 45S rDNA locus located at chromosome 2 (this work) made possible the differentiation of the parental origin of these chromosomes in the diploid and tetraploid hybrid plants analyzed (Figure 1). Images were captured using an Olympus BX-60 microscope with an Olympus DP71 camera and processed with Adobe Photoshop CS5 software.

## Results

### Chiasma Analyses in Diploid Plants of Col, L*er* and Col/L*er* Hybrids

**FIGURE 2:**
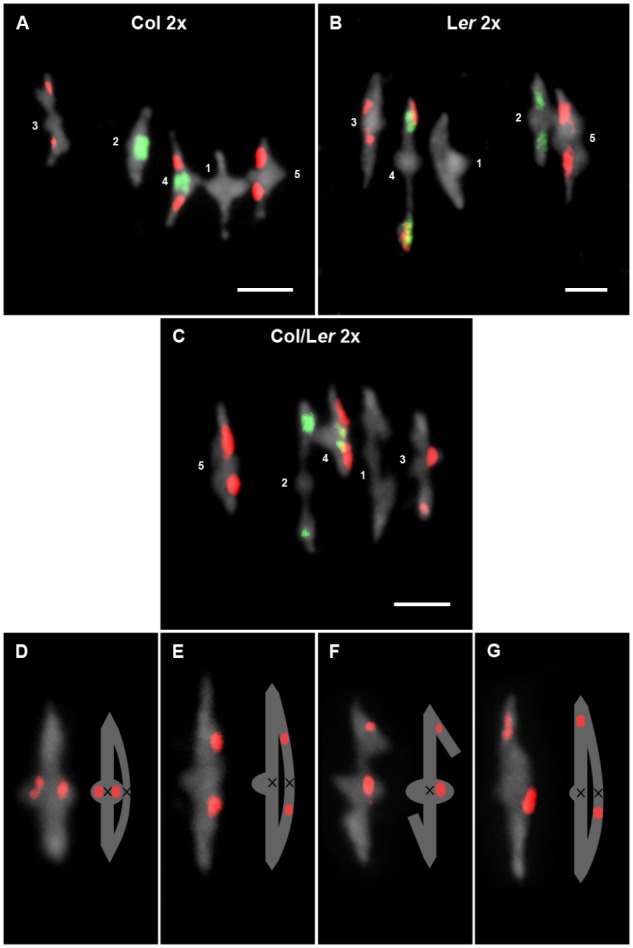
Cytological analysis of diploid cells at metaphase I. Representative examples of metaphases I from Col 2x **(A)**, L*er* 2x **(B)**, and Col/L*er* 2x **(C)**. Chromosomes are identified by FISH with 5S (red) and 45S rDNA (green) probes. Images and representations of bivalents formed by chromosomes 3 in which the chiasma (black cross) has been formed before (**D,F**) or after (**E,G**) the position of the 5S rDNA in a L*er* (**D,E**) and in a Col/L*er* (**F,G**) background. Bars represent 5 μm.

Chromosome morphology together with 45S (NOR) and 5S rDNA FISH probes allow the identification of the whole complement set of *Arabidopsis* in some accessions (Fransz et al., 1998; Sanchez-Moran et al., 2002). Chromosomes 1, 3 and 5 are submetacentric/metacentric, while chromosomes 2 and 4 are acrocentric. Chromosomes 1 do not possess any rRNA genes. Chromosomes 2 are characterized by the presence of 45S rDNA sequences distally on their short arms. Chromosomes 3 and 5 bear 5S rRNA genes and chromosomes 4 have both 45S and 5S rDNA sequences also located on the short arm. Col and L*er* are distinguished because 5S rRNA genes are located on a different arm at chromosome 3 (short arm in Col and long arm in L*er*). In this study, we have also detected another difference between both accessions. The NOR region belonging to chromosome 2 is bigger in Col than in L*er* (Figures 1, 2A–C).

Chiasma scoring was conducted in PMCs at metaphase I. Three plants were analyzed in each accession and also in the hybrid progeny obtained from the cross between both accessions. Since there were no significant differences in the mean chiasma frequencies per cell among them, individual plant data were grouped. In all of the cells assessed in this study, the five chromosome pairs invariably formed five bivalents that could be classified into two categories: rod and ring. A rod (open) bivalent has a single chiasma, whereas in a ring (close) bivalent both chromosome arms are bound by chiasmata.

The mean chiasma frequencies per bivalent and per cell are summarized in Table 1. Col showed a significantly higher mean chiasma frequency per cell (10.20 ± 0.14) than L*er* (9.13 ± 0.10; *t* = 6.2, *p* < 0.001). The value for the Col/L*er* hybrid was intermediate to the previous ones (9.48 ± 0.11; *n* = 120), and it was statistically significant respect to both Col (*t* = 4.14, *p* < 0.001) and L*er* (*t* = 2.39, *p* < 0.05). In all the backgrounds analyzed, individual bivalent chiasma frequencies changed according to the chromosome size (the chromosome 1 had the highest mean chiasma frequency while the short acrocentric chromosomes, 2 and 4, presented the lowest frequencies).

**Table 1:** Chiasma frequencies observed for the different chromosomes (1–5) in PMCs from Col, L*er*, and Col/L*er* diploid plants.

	Chromosomes					
		
	1	2	3	4	5	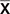	*N*
Col 2x	2.55 (25.0)	1.73 (17.0)	2.16 (21.2)	1.50 (14.7)	2.28 (22.4)	10.20	70
L*er* 2x	2.09 (22.9)	1.74 (19.1)	1.79 (19.6)	1.61 (17.6)	1.90 (20.8)	9.13	158
Col/L*er* 2x	2.14 (22.6)	1.76 (18.6)	1.85 (19.5)	1.70 (17.9)	2.04 (21.5)	9.48	120

*Values showed in parentheses represent the contribution in percentage of each chromosome to the total mean chiasma frequency (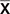). Number of cells analyzed (N).*

In L*er*, the interstitial 5S rDNA region on chromosome 3 divides the long arm of this chromosome in two regions: a proximal region between the centromere and the 5S rRNA genes, and a distal region from these genes to the telomere. This feature has allowed a more accurate analysis of chiasma distribution on this arm not only in this accession but also in the Col/L*er* hybrid (Figures 2D–G). In both backgrounds, about 50% of chiasmata were located in each region (L*er*: χ_1_^2^ = 1.58, *p* > 0.05; Col/L*er*: χ_1_^2^ = 1.09, *p* > 0.05). Therefore, chiasma localization on this chromosome arm do not change in the hybrid.

### Multivalent and Chiasma Analyses in Autotetraploid Plants of Col, L*er* and Col/L*er* Hybrids

**FIGURE 3:**
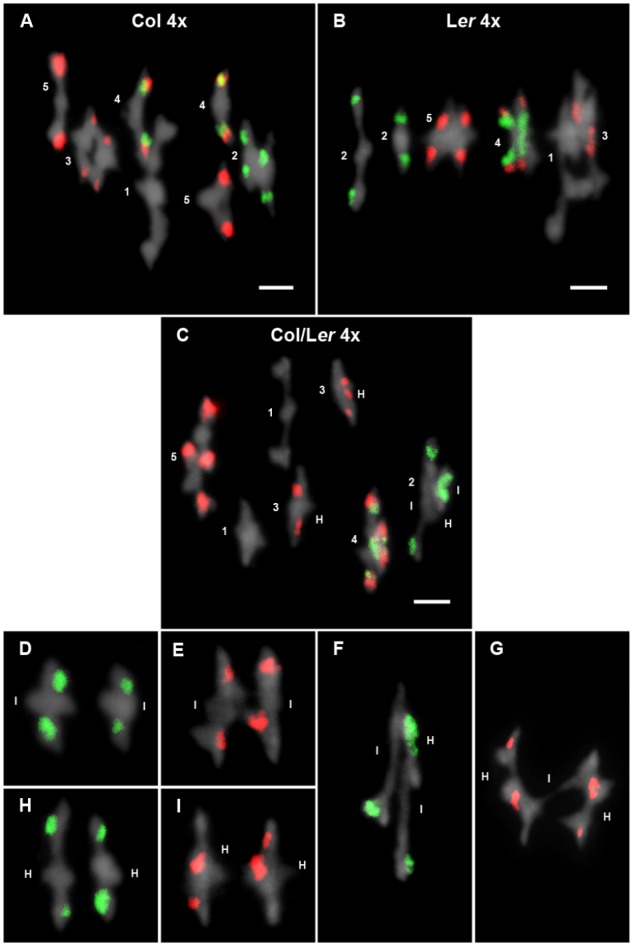
Cytological analysis of autotetraploid cells at metaphase I. Representative examples of metaphases I from Col 4x **(A)**, L*er* 4x **(B)**, and Col/L*er* 4x **(C)**. Chromosomes are identified by FISH with 5S (red) and 45S rDNA (green) probes. In Col 4x, tetrasomes 1, 2, and 3 appear as quadrivalents, whereas two bivalents are formed in tetrasomes of chromosomes 4 and 5. In L*er* 4x, only the tetrasome of chromosomes 2 appears as two bivalents. In the hybrid, two bivalents are formed in tetrasomes of chromosomes 1 and 3, whereas the remaining tetrasomes appear as quadrivalents. The figure also includes examples of associations between identical chromosomes in bivalents formed by chromosomes 2 **(D)** and chromosomes 3 **(E)**. Examples of associations between homologous chromosomes in bivalents formed by chromosomes 2 **(H)** and chromosomes 3 **(I)**. Examples of quadrivalents formed by chromosomes 2 **(F)** and 3 **(G)**. Capital letters I and H represent identical and homologous association, respectively. Bars represent 5 μm.

Frequencies for the different configurations observed at metaphase I were recorded for each chromosome in three plants of each genotype (Table 2). Data from plants sharing the same background were grouped since there were no significant differences in multivalent and chiasma frequencies among them. Chromosomes were predominantly associated as bivalents, quadrivalents, and trivalent + univalent (Figures 3A–C). Since the frequency of the latter was very low (16/186 = 9% in Col 4x; 8/50 = 16% in L*er*; 11/139 = 8% in Col/L*er* 4x), no distinction was made between quadrivalents and trivalents and they were simply grouped as multivalents (Table 2).

**Table 2:** Multivalents (M), bivalent pairs (II) and chiasma frequency (Xta) observed for the different chromosomes (1–5) in PMCs from Col, L*er*, and Col/L*er* autotetraploid plants.

		Chromosomes					
			
		1	2	3	4	5	Total	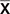	*N*
Col 4x	M	153 (82.3)	129 (69.4)	150 (80.6)	122 (65.9)	151 (81.2)	705 (75.9)	3.79	186
	II	33 (17.7)	57 (30.6)	36 (19.4)	63 (34.1)	35 (18.8)	224 (24.1)	1.20	
	Xta	4.19 (21.0)	4.02 (20.1)	3.91 (19.6)	3.82 (19.1)	4.05 (20.2)		19.99	
L*er* 4x	M	40 (80)	31 (64.6)	36 (73.5)	33 (70.2)	39 (73.6)	179 (72.5)	3.58	53
	II	10 (20)	17 (35.4)	13 (26.5)	14 (29.8)	14 (26.4)	68 (27.5)	1.36	
	Xta	4.00 (21.6)	3.60 (19.5)	3.64 (19.7)	3.54 (19.1)	3.72 (20.1)		18.50	
Col/L*er* 4x	M	97 (69.8)	70 (51.5)	70 (50.7)	89 (64)	99 (71.2)	425 (61.5)	3.06	139
	II	42 (30.2)	66 (48.5)	68 (49.3)	50 (36)	40 (28.8)	266 (38.5)	1.91	
	Xta	4.07 (21.4)	3.78 (19.9)	3.60 (18.9)	3.59 (18.9)	3.99 (20.9)		19.03	


*Values showed in parentheses represent the percentage of multivalents and pairs of bivalents to the total cells (N), and the contribution of each chromosome to the total mean chiasma frequency (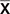). Number of cells analyzed (N).*

Synaptic configurations in autotetraploids with metacentric chromosomes have usually been estimated under the following premises (for review see Sybenga, 1975): (i) the presence of two independent synapsis initiation points per chromosome, one at each end; (ii) the absence of synapsis preferences; (iii) the existence of same probabilities for chiasma formation in all meiotic configurations; and (iv) the possibility of free partner switches between the two synapsis initiation points at each chromosome. In this context, there are nine possible configurations to be formed among each group of homologous chromosomes (tetrasome), of which six are quadrivalents (2/3) and the remaining three are pairs of bivalents (1/3), i.e., the ratio of multivalents to bivalent pairs at prophase I would be 2:1.

The observed ratios of multivalents to bivalent pairs were tested for the agreement with the theoretical 2:1 ratio for each chromosome in the three autotetraploid genotypes analyzed (Tables 2, 3). The level of multivalent formation over the five chromosomes (705 multivalents: 224 bivalent pairs) displayed by Col 4x significantly excess the 2:1 ratio (66.66% multivalents) [χ_(1)_^2^ = 35.55; *p <* 0.001]. In this accession, at the chromosomal level, only the three largest chromosomes of the complement (1, 3 and 5) showed multivalent frequencies consistently in excess of the 66.66%. L*er* 4x also presented an excess of multivalents (72.5%), but with a value that is at the limit of the significance level [χ_(1)_^2^ = 3.74; *p* = 0.053]. In this case, only chromosome 1 showed a significant excess of multivalents, while the other four chromosomes fitted to the random theoretical expectations. Conversely, there was a significant excess of bivalent pairs in the Col/L*er* 4x hybrid (38.5%) [χ_(1)_^2^ = 8.28; *p* < 0.01], mainly due to the behavior of chromosomes 2 and 3 (Table 3).

**Table 3:** Chi-square test values (*χ_1_^2^*) testing goodness of fit to 2:1 ratio of multivalents: bivalent pairs for the different chromosomes (1–5) in PMCs from Col, L*er* and Col/L*er* autotetraploid plants.

	Chromosomes					
	
	1	2	3	4	5	Total
Col 4x	> 20.35^∗∗∗^	= 0.60^NS^	> 16.35^∗∗∗^	= 0.04^NS^	> 17.64^∗∗∗^	> 35.55^∗∗∗^
L*er* 4x	> 4.00^∗^	= 0.09^NS^	= 1.02^NS^	= 0.64^NS^	= 0.64^NS^	= 3.74^NS^
Col/L*er* 4x	= 0.61^NS^	< 14.13^∗∗∗^	< 15.78^∗∗∗^	= 0.44^NS^	= 1.30^NS^	< 8.28^∗∗^


*Less than and greater than symbols indicate direction of deviation: < ( < 2:1), > ( > 2:1). ^∗^p ≤ 0.05; ^∗∗^p ≤ 0.01; ^∗∗∗^p ≤ 0.001; NS, not significant.*

Col 4x showed the highest mean cell chiasma frequency (19.99 ± 0.11) followed by the hybrid Col/L*er* 4x (19.03 ± 0.15). L*er* 4x presented the lowest frequency (18.5 ± 0.27) (Table 2). These means are about twofold the corresponding mean cell chiasma frequencies of the diploid counterparts. There were significant differences between the means of Col 4x and L*er* 4x (*t* = 5.19, *p* < 0.001), and also between Col 4x and the hybrid 4x (*t* = 3.24, *p* < 0.001), but not between L*er* 4x and the hybrid 4x (*t* = 1.19, *p* = 0.08). The relative contribution of each chromosome to the total mean cell chiasma frequency was similar in these three backgrounds, with the exception of a slightly lower contribution of chromosome 3 in the hybrid (Table 2). In addition, chiasma localization was analyzed in the long arm of chromosome 3 in L*er* 4x and also in the hybrid 4x, but only in cells in which chromosomes 3 did not form a multivalent. As well as in diploid cells, about 50% of chiasmata were located in the proximal region (centromere – 5S rDNA) and the remaining 50% in the distal region (5S rDNA – telomere).

### Competition for Chiasma Formation Between Identical and Homologous Chromosomes in the Hybrid Col/L*er* 4x

One of the main objectives of this study was to analyze whether chromosome intraspecific differences in autotetraploid plants are enough to determine preferences in terms of chiasma formation. Taking into account the cytological differences between Col and L*er* accessions, namely: variations in the size of the NOR region located on the short arm of chromosome 2 and changes in the localization of the 5S rDNA at chromosome 3, Col/L*er* diploid hybrids were treated with colchicine to obtain autotetraploid plants. In these hybrids, there is one pair of identical chromosomes from Col and another identical pair from L*er*. These two pairs of identical chromosomes are homologous, but non-identical with each other (Figure 1). Then, two types of two-by-two metaphase I associations are possible for any chromosome arm: between identical chromosomes or between homologous chromosomes (Figures 3D–I). Assuming that chiasma formation takes place randomly among the four members of each tetrasome, homologous associations will be twice as common as identical ones.

Following the criteria established by Benavente and Orellana (1991), in this analysis we have included cells with at least one chiasma between identical or homologous chromosomes (regardless of the chromosome configuration adopted by the corresponding tetrasome) to test randomness (Figures 3D–I and Table 4). When data from multivalents and bivalent pairs were grouped, we detected a different behavior of chromosomes 2 and 3. We observed random chiasma formation between identical and homologous arms of chromosome 2, and homologous preferences for chiasma formation in both arms of chromosome 3. This tendency was also maintained when only data from bivalent pairs were considered, although in this case the excess of chiasmata between homologous short arms of chromosome 3 was at the limit of the significance level.

**Table 4:** Number of Col/L*er* 4x chromosome configurations with at least one chiasma between identical (I) or homologous (H) chromosomes in bivalent pairs (II) and multivalents + bivalent pairs (M+II) and the goodness of fit to the expected ratio 1I:2H.

	Chromosome 2						Chromosome 3					
		
	Short arm			Long arm			Short arm			Long arm		
				
	I	H	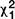	I	H	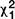	I	H	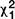	I	H	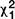
M+II	36	63	0.41	34	67	0.01	21	78 ↑	6.58^∗^	20	85 ↑	9.64^∗^
II	24	42	0.27	22	44	0.00	15	51	3.34	14	54 ↑	4.97^∗^


*Chi-square test values obtained (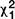). Values that exceed the 1:2 (I:H) ratio expected at random (↑). Chi-square test values statistically significant, ^∗^p ≤ 0.05.*

## Discussion

### Multivalent and Chiasma Frequencies at Metaphase I

The frequencies of multivalents observed in Col 4x plants significantly exceed the 2:1 ratio (66.66% multivalents) expected on the random-end pairing model (Table 3), which means that, despite their small size, at least in this accession chromosomes have more than two autonomous synaptic initiation sites (López et al., 2008), and more than one synaptic partner switch per tetrasome. In addition, there was a significant excess of bivalent pairs in the autotetraploid Col/L*er* hybrid (Table 2; χ_1_^2^ = 8.30, *p* < 0.01). It might be produced, at least partially, as a consequence of the heterozygosity.

Col and L*er* diverged ∼200,000 years ago (Koch et al., 2000). Around 16,000 single feature polymorphisms between Col and *Ler* accessions were detected in ∼8,000 of the ∼26,000 genes represented in a 44,000 feature exon-specific oligonucleotide array (Singer et al., 2006). Furthermore, more than 6,000 insertions or deletions distinguish both accessions, which differ in 564 transpositions and 47 inversions that comprise around 3.6 Mb (Ziolkowski et al., 2009; Zapata et al., 2016). Increases in bivalent frequency are strongly chromosome dependent and are generally ascribed to overall decreases in chiasma frequency and/or changes in chiasma distribution, with a more rapid response of the shortest chromosomes to these alterations. The behavior of chromosome 3 in L*er* can shed some light to this issue since it carries a 170 kb inversion on the short arm (Zapata et al., 2016). Hence, Col/L*er* hybrid is heterozygous for such inversion, and it is well known that the heterozygosity for inversions suppresses meiotic recombination.

The mean cell chiasma frequencies of chromosome 3 in L*er* 4x and Col/L*er* 4x are similar (3.64 vs. 3.60; *t* = 0.31, *p* = 0.76), but there are significant differences between the means of bivalent pairs (0.26 vs. 0.49; *t* = 3.03, *p <* 0.001) (Table 2). Hence, other factors in addition to chromosome rearrangements, such as genotypic, epigenetic or cryptic structural differences along chromosomes, may be involved in the increase of bivalent frequency observed not only in this hybrid but also, for instance, in established autotetraploid lines of *Arabidopsis* (Santos et al., 2003). On these grounds, Zhang et al. (2015) reported that, after 49 self-pollinated generations, autotetraploid rice showed a significant increase in the methylation of class II transposons in relation to its diploid donor that may affect gene expression. Also, Dar et al. (2017) observed differences between the frequency of both quadrivalents and bivalents from C_0_ to C_2_ synthetic autotetraploids of *Phlox drummondii*, associated with changes in both repetitive and non-repetitive regions.

Polyploidy is a major process in plant speciation. The potential evolutionary success of polyploids has been linked, among other hypotheses, to the buffering of mutations and sub- and neo-functionalization of duplicated genes (see for reviews, Otto, 2007; Soltis and Soltis, 2009; Parisod et al., 2010; Zielinski and Mittelsten Scheid, 2012). It has been reported that polyploids of *Gossypium* and *Arabidopsis* enhance meiotic recombination compared with diploids (Desai et al., 2006; Pecinka et al., 2011). Increases in chiasma frequency could help to the establishment of new polyploid species by rapid creation of genetic diversity when population sizes are small. The data reported in the present work unfit to this proposal since the autotetraploids showed chiasma frequencies about twofold in comparison with their diploid counterparts (Tables 1, 2). However, the possibility to obtain an increase in recombination in certain chromosome regions cannot be ruled out. It would be interesting to test this hypothesis by examining chiasma frequencies not only in other *Arabidopsis* accessions but also in other non-related species.

### Competition for Chiasma Formation Among Identical and Homologous Chromosomes

Benavente and Orellana (1991) analyzed preferences for chiasma formation in synthetic autotetraploids of *Secale cereale* obtained from heterozygous hybrids for telomeric C-bands at chromosome 1R. They found a clear tendency for preferences between identical partners in inter-subspecific hybrids. This tendency increased in inter-specific hybrids with a higher chromosomal divergence between homologous chromosomes. These results reflect the potential effect of chromosomal differentiation on chiasma preferences in polyploids (see also Jenkins and Chatterjee, 1994). However, the hybrids resulting from crosses between inbred lines showed a wide range of preferential associations. Therefore, chiasmata between identical partners are not always favored.

In this study, we have observed that although chromosomes 2 and 3 exhibited similar frequencies of bivalent pairs (0.47 and 0.49, respectively) and chiasmata (3.78 and 3.60, respectively) (Table 2), they presented different preferences in chiasma formation in the hybrid Col/L*er* 4x. Chiasmata were randomly formed between identical and homologous chromosomes 2, but preferentially established between homologous chromosomes 3 (Table 4). These results indicate that although chromosome differentiation between related genomes may be the main cause of the excess of bivalents in the hybrid, bivalent formation between identical chromosomes is not necessarily favored (Sybenga, 1992, 1994; Bourke et al., 2015). In this regard, random chiasma formation among identical and homologous chromosomes 2 could be related to their close spatial nuclear location as a consequence of bearing the NOR region on the short arm, since differences in the number of 45S tandem repeats (Rabanal et al., 2017) do not seem to have an influence. On the other hand, preferences for chiasma formation between homologous chromosomes 3 could be more related to specific features of particular chromosome regions. Actually, in L*er* the 5S rDNA region on the long arm of this chromosome is close (∼6 Mb) to the 170 kb inversion mentioned before (Simon et al., 2018). This means that the genomes of the two accessions are different in a large region, which would have important consequences for meiotic recombination. However, recent meiotic recombination analysis suggests that high levels of sequence divergence are not necessarily inhibitors of meiotic recombination (Barth et al., 2001; Singer et al., 2006; Salomé et al., 2012). This idea is in agreement with a positive correlation of ancestral recombination frequencies and regions with high sequence divergence (Kim et al., 2007). In addition, heterozygous regions increase chiasma formation when are juxtaposed with homozygous regions, which reciprocally decrease (Ziolkowski et al., 2015). In relation to chromosome 3, Barth et al. (2001) found a strong negative correlation between genetic similarities of ecotypes and recombination frequencies for two adjacent markers located on the long arm of this chromosome, but not for other genomic regions. In general, there are difficulties in mapping and sequencing this chromosome, consequently this fact suggests the existence of unusual chromatin-related features respect to the other chromosomes of the complement (Schmidt, 2018).

Taking into account the information compiled in this work, it is evident that when the chromosome complement of a diploid individual is duplicated, the degree of relationship between two chromosomes within each tetrasome may be greater than mere homology. In this situation, there is a competition for chiasma formation between identical and homologous chromosomes that can be resolved through different ways depending on the chromosome. Accordingly, identical and homologous chromosome regions will persist in each tetrasome in a differential pattern throughout generations. This chromosomal genetic variation has not be considered in current models about tetrasomic and disomic inheritance and it could produce a relevant impact on haplotypes.

## Author Contributions

PP-N completed the experiments and performed the data analyses. All authors conceived and designed the experiments, wrote and reviewed the manuscript.

## Conflict of Interest Statement

The authors declare that the research was conducted in the absence of any commercial or financial relationships that could be construed as a potential conflict of interest.
